# Association of TyG index with prostate-specific antigen (PSA) in American men: results from NHANES, 2003–2010

**DOI:** 10.1007/s11845-023-03431-5

**Published:** 2023-06-20

**Authors:** Mengyu Zhang, Jiankang Zhang, Zengshu Xing

**Affiliations:** Department of Urology, Affiliated Haikou Hospital of Xiangya Medical School, Central South University, Haikou, 570208 China

**Keywords:** Cross-sectional study, NHANES, Population-based study, PSA, Triglyceride–glucose index

## Abstract

**Background:**

In recent years, triglyceride-glucose index (TyG) was a new indicator of insulin resistance, and it has been widely reported that it may be associated with serum prostate-specific antigen (PSA) concentrations.

**Aims:**

We intended to investigate the possible connection between serum PSA concentration and the TyG index.

**Methods:**

This is a cross-sectional study of adults with complete data on TyG and serum PSA concentrations (ng/ml) from the NHANES, 2003–2010. The TyG index is obtained by the formula below: TyG = Ln [triglycerides (mg/dL) × fasting glucose(mg/dL)/2]. Multivariate regression analysis and subgroup analysis were used to examine the connection between the TyG index and serum PSA levels.

**Results:**

Multiple regression analysis of the weighted linear model showed that individuals with a higher TyG index had lower PSA levels. Subgroup analyses and interaction tests showed no apparent dependence on age, race/ethnicity, BMI, household income ratio, education level, and marital status on this negative association (all interactions *p* > 0.05).

**Conclusions:**

TyG index is related to lower serum PSA concentrations in adult men from the USA. Further comprehensive prospective studies are needed to confirm our findings.

## Introduction

Prostate cancer ranks second to lung cancer as the second leading cause of malignancy death in men [1]. Prostate cancer has no specific clinical symptoms at the beginning of the disease. Prostate-specific antigen (PSA) testing is the primary screening for prostate cancer, which can effectively achieve early prostate cancer diagnosis and treatment [2]. However, PSA values have high sensitivity but low specificity. Many studies have suggested that the PSA level may be impacted by various other reasons [3–5]. Since PSA values are used to screen prostate cancer, approximately 50% of diagnosed prostate cancers are overdiagnosed and overtreated [6].

Recent studies have shown a potential link between several abnormal metabolisms and the risk of prostate cancer [7–10]. Many types of research have illustrated that men with diabetes have decreased PSA concentrations compared to those without diabetes [11]. Insulin resistance is key to the status of type II diabetes [12]. It may precede elevated glucose and impaired glucose regulation, and the TyG index has emerged as a new indicator of resistance to insulin in recent years [13]. Research has elucidated that insulin resistance may be related to serum PSA concentrations [14]. However, the correlation between the TyG index and PSA remains unknown. Therefore, our research was designed to investigate the correlation between the TyG index and PSA concentrations in individuals from National Health and Nutrition Examination Survey (NHANES).

## Methods the source of data and study population

NHANES uses complex, multi-stage, probability sampling methods to provide information about different US populations or health topics. The NHANES protocol received approval from the National Center for Health Statistics Study’s Committee on Ethical Review, and all survey participants signed the documents of informed consent. Survey data were obtained from the NHANES 2003–2010.

**Fig. 1:**
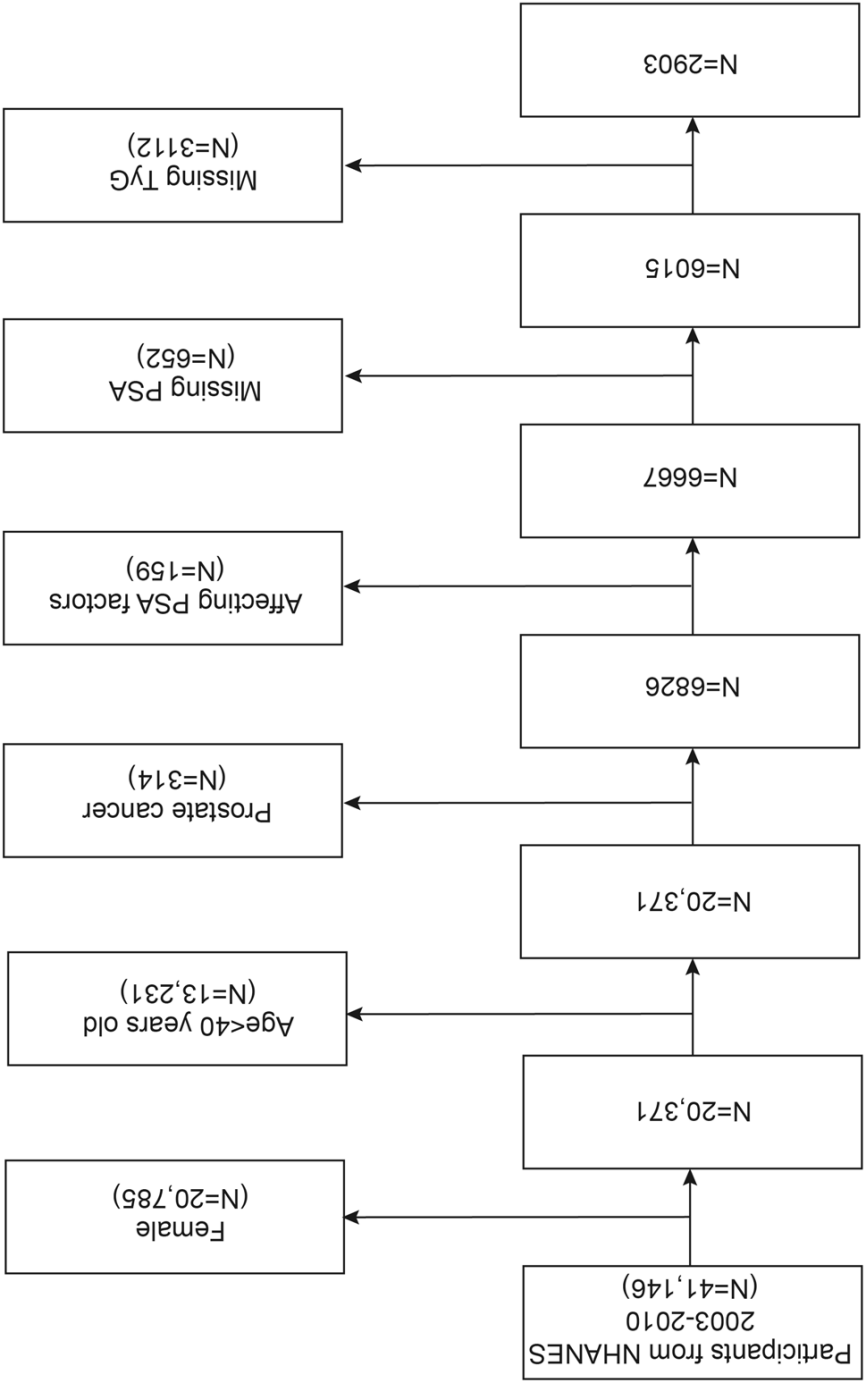
The flowchart in selecting the studying participants

We excluded 20,785 female subjects, 13,231 subjects aged < 40 years, 314 subjects with a diagnosis of prostate cancer, and 652 subjects with a possible effect on PSA levels that were excluded: (1) the presence of prostate infection or inflammation, (2) prostate biopsy within a week, and (3) urological surgery and examination within 1 month. and 3764 subjects who lacked PSA and TyG index. The study ultimately included 2903 eligible participants (Fig. 1).

### Research variables

The independent variable in this research was the TyG index, and the dependent variable was the total PSA (ng/ml). In this study, we used the TyG index as the exposure variable, and the TyG index is obtained by the formula below: TyG = Ln [triglycerides (mg/dL) × fasting glucose (mg/dL)/2] [15]. We selected the following covariates. Variables comprised age, waist circumference (cm), BMI (kg/m^2^), household income to poverty ratio (PIR), HDL-C (mg/dL), LDL-C (mg/dL), total cholesterol (TC) (mg/dL), C-reactive protein (CRP) (mg/dL), and glycohemoglobin (%), race, education level, smoking status, drinking status, diabetes history, hypertension history, education level, and marital status. For more detailed information on these covariates, please visit the official NHANES website.

### Statistical analysis

We analyzed the TyG index and total PSA levels statistically according to the standards of the CDC and Prevention guidelines (https://wwwn.cdc.gov/nchs/nhanes/tutorials/default.aspx). We divided the TyG index into four groups according to their quartile-taking values and described them with a weighted linear regression model. Weighted univariate and multiple linear regression models were used to identify the β values and 95% confidence intervals calculated for the analysis of the TyG index and PSA concentration. Three statistical models were constructed: model 1, with no covariate adjustments; model 2, adjusted for age and race data only; and model 3, adjusted for all covariates. A smooth curve fit was constructed by adjusting for covariates. We used a weighting approach to reduce the significant volatility of the data set. All statistical analyses were carried out using the applications EmpowerStats (http://www.empowerstats.net/cn/index.php) and R 4.2.1 (https://www.r-project.org/). A *P*-value of < 0.05 was believed to be Statistical meaning in all analyses.

## Result baseline population characteristics

A total of 2903 people took part in this research, and the average age of the participants was 55.80 years (± 11.40). Then, we categorized the various TyG indices into interquartile (Q1–Q4). Compared to participants with higher levels of TyG index, subjects with lower TyG index were older, had lower LDL-C, higher HDL-C, lower total cholesterol, lower waist size and BMI, a higher degree of education, and a lower incidence of hypertension and diabetes. In contrast, participants with a higher TyG index had lower PSA concentrations and higher glycohemoglobin; they were more likely to smoke at least 100 cigarettes. Most participants were non-Hispanic white (Table 1).

**Table 1:** Baseline characteristics of the selected participants

**TyG**	**Q1**	**Q2**	**Q3**	**Q4**	***P*-value**
	***N* = 725**	***N* = 726**	***N* = 726**	***N* = 726**	
**PSA ng/ml**	1.66 ± 2.84	1.58 ± 2.73	1.44 ± 1.86	1.33 ± 1.95	0.0457
**Sociodemographic variables**					
**Age, mean ± SD (years)**	55.58 ± 11.94	55.50 ± 11.16	57.14 ± 11.47	55.06 ± 10.92	0.0038
**Poverty to income ratio, mean ± SD (years)**	3.35 ± 1.57	3.40 ± 1.57	3.29 ± 1.55	3.27 ± 1.60	0.3970
**Race/ethnicity (%)**					< 0.0001
**Mexican American**	4.18	4.93	7.02	8.64	
**Other Hispanic**	2.33	2.40	4.12	4.52	
**Non-Hispanic White**	76.11	78.13	77.16	74.07	
**Non-Hispanic Black**	13.14	8.32	6.99	5.27	
**Other race/ethnicity**	4.23	6.23	4.71	7.49	
**Education (%)**					0.0005
**Less than high school**	18.90	16.41	20.01	22.06	
**High school**	22.82	24.31	21.46	28.77	
**More than high school**	58.28	59.29	58.5454.86	49.18	
**Marital status (%)**					0.0204
**Married**	70.09	73.41	75.55	70.09	
**Single**	25.21	20.28	21.07	25.02	
**Living with a partner**	4.70	6.30	3.38	4.89	
**Variables of laboratory data**					
**LDL-C, mean ± SD (mg/dL)**	114.45 ± 32.86	123.59 ± 31.58	122.25 ± 37.79	116.09 ± 36.01	< 0.0001
**HDL-C, mean ± SD (mg/dL)**	59.28 ± 16.29	51.41 ± 13.01	45.66 ± 10.61	40.26 ± 9.11	< 0.0001
**Total cholesterol, mean ± SD (mg/dL)**	187.48 ± 36.76	196.56 ± 35.20	198.99 ± 40.76	212.68 ± 47.33	< 0.0001
**Glycohemoglobin (%)**	5.45 ± 0.54	5.53 ± 0.55	5.67 ± 0.82	6.24 ± 1.52	< 0.0001
**C-reactive protein, mean ± SD (mg/dL)**	0.37 ± 0.97	0.32 ± 0.77	0.52 ± 1.56	0.37 ± 0.46	0.0015
**Medical examination and personal life history**					
**Body mass index, mean ± SD (Kg/m2)**	36.97 ± 37.92	38.59 ± 38.18	41.20 ± 39.86	44.31 ± 42.27	0.0031
**Waist circumference, mean ± SD (cm)**	94.43 ± 21.31	97.70 ± 21.61	100.72 ± 23.30	103.10 ± 24.84	< 0.0001
**Smoked at least 100 cigarettes in life**					0.0270
**Yes**	54.82	58.54	57.94	62.66	
**No**	45.18	41.46	42.06	37.34	
**Had at least 12 alcohol drinks 1 year**					0.3660
**Yes**	82.73	84.30	85.13	81.86	
**No**	17.27	15.70	14.87	18.14	
**Comorbidities (%)**					
**Hypertension history**					< 0.0001
**Yes**	32.26	37.49	43.48	52.53	
**No**	67.74	62.51	56.52	47.47	
**Diabetes history**					< 0.0001
**Yes**	5.18	7.30	12.03	23.35	
**No**	93.43	90.79	85.08	72.85	
**Borderline**	1.39	1.92	2.89	3.81	

Values are means ± SD or percentages. All estimates were weighted to be nationally representative

### Relationship between PSA concentration and TyG index

In the unadjusted model, each single unit increment in the TyG index was correlated with a 0.20-ng/mL reduction in PSA levels. [*β* =  − 0.20, 95% CI: (− 0.33, − 0.06), *P* < 0.05].

In models partially adjusted for race/ethnicity and age, the PSA concentration decreased by 0.17 ng/mL for each unit increase in the TyG index. [*β* =  − 0.17, 95%CI: (− 0.30, − 0.04), *P* < 0.05]. For sensitive analysis, the TyG index was changed from a continuous variable to a categorical variable (quartiles). Compared to Q1, subjects in Q4 had a statistically significant 28% decrease in PSA concentration. [*β* =  − 0.28, 95%CI: (− 0.52, − 0.04), *P* < 0.05]. In fully adjusted models adjusted for age, race/ethnicity, education level, poverty income ratio, marital status, LDL-C, HDL-C, TC, CRP, glycohemoglobin (%), BMI (kg/m^2^), waist circumference (cm), smoking status, drinking status, history of hypertension, and history of diabetes, each increase of one unit of TyG index was linked with a 0.57-ng/mL reduction in PSA level (Table 2).

**Table 2:** Univariate and multivariate analyses by the weighted linear model

**Exposure**	**Non-adjusted model**	**Incomplete adjusted model**	**Fully adjusted model**
TyG	− 0.20 (− 0.33, − 0.06) 0.0040	− 0.17 (− 0.30, − 0.04) 0.0106	− 0.57 (− 1.05, − 0.09) 0.0207
TyG			
Q1	Ref	Ref	Ref
Q2	− 0.08 (− 0.32, 0.16) 0.5161	− 0.06 (− 0.29, 0.18) 0.6304	− 0.18 (− 0.46, 0.11) 0.2182
Q3	− 0.22 (− 0.47, 0.03) 0.0843	− 0.31 (− 0.55, − 0.06) 0.0130	− 0.31 (− 0.68, 0.06) 0.0988
Q4	− 0.33 (− 0.58, − 0.08) 0.0096	− 0.28 (− 0.52, − 0.04) 0.0230	− 0.38 (− 0.99, 0.22) 0.2097
*p *for trend	0.0050	0.0070	0.1318

Non-adjusted model adjusts for: None. Incomplete adjusted model adjust for: age (years); sex; race/ethnicity (%). Fully adjusted model adjust for: age (years); race/ethnicity; education level; poverty income ratio; marital status; LDL-C; HDL-C; total cholesterol; C-reactive protein; glycohemoglobin (%); BMI (kg/m^2^); waist circumference (cm); at least 100 cigarettes in lifetime; at least 12 alcoholic drinks in a year; history of hypertension; history of diabetes mellitus

### Subgroup analysis

To study the relationship between the TyG index and PSA concentration in different populations, we conducted subgroup analyses stratified by age, race/ethnicity, education level, marital status, BMI, and PIR. PSA concentrations were likely to be lower for those aged > 70 years, non-Hispanic whites, the educational level below high school, married status, BMI between 25 and 28, and higher PIR, and the negative connection between the TyG index and PSA concentration was notable (*P* < 0.05). In addition, interaction tests did not show statistically significant variations in the association between TyG index and PSA concentration between strata, indicating that age, race/ethnicity, education level, marital status, BMI, and PIR were not significantly dependent on this negative association (all interactions *P* > 0.05) (Table 3).

**Table 3:** Analysis of TyG index with PSA concentration

**TyG index**	***β***	**95% CI**	***p*-value**	***p* for interaction**
**Stratified by age**				0.1179
**<** **50**	− 0.19	(− 0.79, 0.41)	0.5370	
**50–70**	− 0.65	(− 1.18, − 0.11)	0.0179	
**> 70**	− 0.77	(− 1.49, − 0.05)	0.0357	
**Stratified by race**				0.1472
**Mexican American**	− 0.04	(− 1.11, 1.02)	0.9366	
**Other Hispanic**	0.38	(− 1.10, 1.87)	0.6113	
**Non-Hispanic White**	− 0.71	(− 1.23, − 0.20)	0.0067	
**Non-Hispanic Black**	0.01	(− 0.79, 0.82)	0.9769	
**Other race/ethnicity**	− 0.63	(− 2.06, 0.81)	0.3924	
**Stratified by education**				0.7820
**Less than high school**	− 0.65	(− 1.28, − 0.03)	0.0406	
**High school**	− 0.45	(− 1.06, 0.15)	0.1428	
**More than high school**	− 0.50	(− 1.01, 0.01)	0.0562	
**Stratified by marital status**				0.1938
**Married**	− 0.88	(− 1.45, − 0.30)	0.0028	
**Single**	− 0.01	(− 0.84, 0.82)	0.9844	
**Living with a partner**	− 0.26	(− 2.13, 1.60)	0.7810	
**Stratified by BMI**				0.0901
** < 25**	− 0.22	(− 1.23, 0.80)	0.6773	
**25–28**	− 1.31	(− 2.18, − 0.45)	0.0030	
** > 28**	− 0.23	(− 0.88, 0.41)	0.4785	
**Stratified by ratio of family income**				0.2017
**Low group**	− 0.38	(− 0.95, 0.20)	0.2016	
**Median group**	− 0.19	(− 0.82, 0.45)	0.5621	
**High group**	− 0.66	(− 1.24, − 0.09)	0.0244	

### Non-linear correlation between PSA concentration and TyG index

**Fig. 2:**
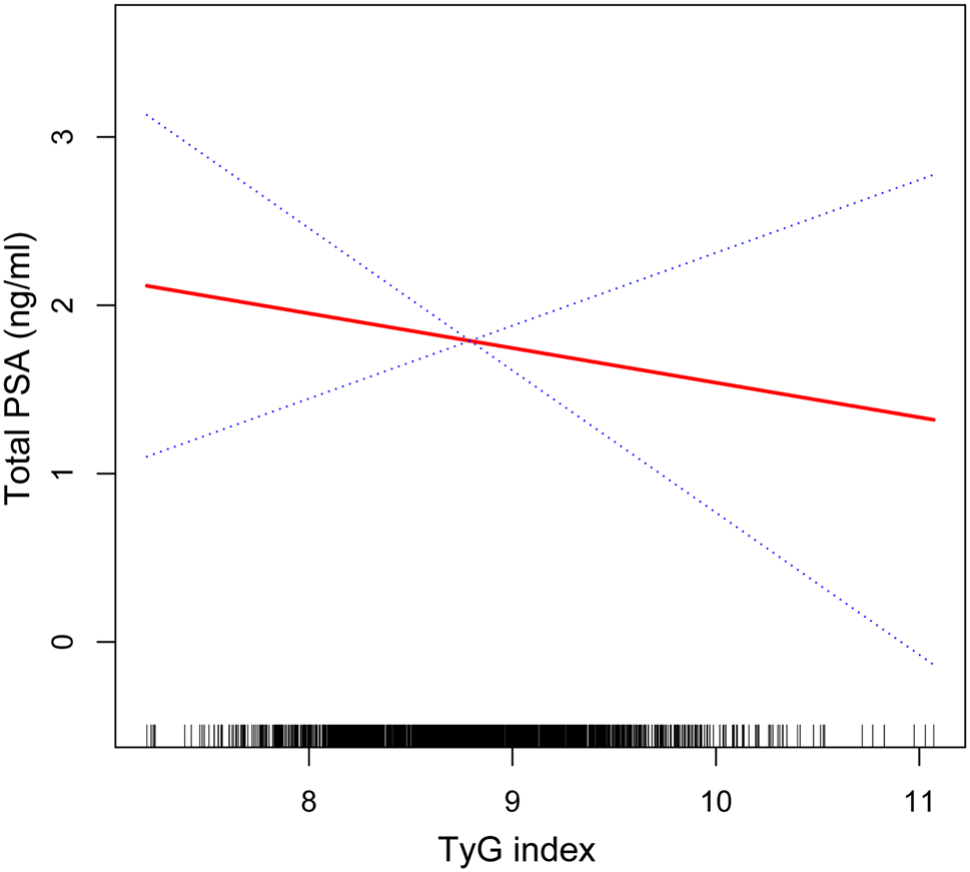
The relationship between serum triglyceride and prostate-specific antigen (PSA) connections

To investigate the possible non-linear correlation between PSA concentration and the TyG index, after constructing a smoothed curve fit by fully adjusting the model, our results reveal no non-linear correlation between the TyG index and PSA concentration (Fig. 2).

## Discussion

In the cross-sectional study of 2903 subjects included, we obtained a negative relation between the index of the TyG and the PSA level. Subgroup analyses and interaction tests revealed that this correlation was similar across population settings. This result indicates that high TyG is an independent predictor of lower PSA. As far as we know, this is the first research to assess the relationship between the index of the TyG and PSA. TyG index has been used as an important biomarker for T2DM and an early indicator of insulin resistance. It has been shown that physical obesity and metabolism of steroid hormones, response to inflammation, and insulin regulation can influence PSA expression [16, 17]. In a review of the relationship between lifestyle and physiological factors and serum PSA concentrations, Parekh et al. revealed a significant negative connection between PSA concentrations and insulin resistance (*P* = 0.04) [18]. The study by Choi et al. obtained similar results [19].

The mechanisms involved in the relationship between TyG index and serum PSA concentration still need further investigation, and there may be some possible reasons as follows.

Firstly, a larger index of the TyG represents the presence of insulin resistance. A cross-sectional study including 506 Chinese individuals showed that a decrease in serum PSA levels was mainly associated with insulin resistance. As insulin resistance increased, it reduced serum PSA levels by 11.3% [14]. TyG index is a new index to assess insulin resistance in recent years. This study is the first to observe a negative correlation between the index of the TyG and serum PSA levels in a large demographic group. Meanwhile, the negative correlation between TyG and PSA concentrations is also present in various subgroup analyses.

Secondly, many studies have suggested that the TyG index is also associated with cardiovascular disease, diabetes, hypertension, BMI, metabolic syndrome, and disorders of lipid metabolism. Fukui et al. investigated men with type 2 diabetes whose serum prostate-specific antigen (PSA) levels are lower than healthy men and found that diabetes is the independent determinant of serum PSA level [11]. Gao et al. investigated the impact of metabolic syndrome on PSA levels and discovered that MetS was related to reduced PSA levels [20]. A study by Wei et al. on the correlation between triglycerides and PSA levels found an independent negative association between serum triglycerides and PSA levels [21]. In our current study, the relationship between high levels of TyG index and lower PSA levels can be observed to be strongest in the fully adjusted model of baseline characteristics. Therefore, the TyG index is an important factor that should be taken into account in screening subjects at risk for prostate cancer by serum PSA to avoid possible overdiagnosis and overtreatment.

There are several advantages to our study when compared with previously published articles. First, our research was based on a large sample of 2903 participants from the NHANES database, which is a data sample based on the US national population with the help of a standard protocol. All analyses in this study considered appropriate NHANES weights to make the research sample more representative, and the authors considered confounding covariates to make the study’s results even more convincing. However, there are limitations to the findings of this study. First, the authors could not obtain clear causal relationships in our study because the NHANES database is a cross-sectional study design. The results of this study are indeed obtained from a nationally representative dataset; the authors used data from the NHANES dataset from consecutive cycles from 2003 to 2010. The authors attempted to analyze the TyG index of serum PSA concentrations using an updated dataset to evaluate the correlation between the index of the TyG and serum PSA concentrations. However, data on serum PSA concentrations were not collected in the updated dataset. Although adjustments were made for some potential covariates, The authors could not entirely avoid the influences of other potential confounding factors, such as drug use and ejaculation [22]. Additionally, we excluded participants who did not meet the inclusion criteria. Therefore, for these populations, our results cannot be interpreted. To conclude, our survey is based on the NHANES database, which is limited to the US population. As a result, the generalizability of the results of this study is limited by geographic location. All of the above perspectives need to be further evaluated and studied in the future.

## Conclusion

A higher TyG index is associated with lower serum PSA concentrations in adult men from the USA. However, further comprehensive prospective research is needed to verify our results.

## Data Availability

The data from the survey are publicly available on the Internet for data users and researchers around the world (www.cdc.gov/nchs/nhanes/).

## Abbreviations

NHANES: National Health and Nutrition Examination Survey
TyG Index: Triglyceride glucose index
PSA: Prostate-specific antigen
LDL-C: Low-density lipoprotein cholesterol
HDL-C: High-density lipoprotein cholesterol
BMI: Body mass index
PIR: Household income to poverty ratio
TC: Total cholesterol
CRP: C-reactive protein
smoking status: Smoking at least 100 cigarettes in a lifetime
drinking status: Alcohol consumption of at least 12 times per year
